# What evidence is there for implicating the brain orexin system in neuropsychiatric symptoms in dementia?

**DOI:** 10.3389/fpsyt.2022.1052233

**Published:** 2022-11-25

**Authors:** Giorgio Bergamini, Preciosa Coloma, Helene Massinet, Michel Alexander Steiner

**Affiliations:** ^1^CNS Pharmacology and Drug Discovery, Idorsia Pharmaceuticals Ltd., Allschwil, Switzerland; ^2^Clinical Science, Global Clinical Development, Idorsia Pharmaceuticals Ltd., Allschwil, Switzerland

**Keywords:** dementia, neuropsychiatric symptoms, behavioral symptoms, orexin, orexin receptor modulators

## Abstract

Neuropsychiatric symptoms (NPS) affect people with dementia (PwD) almost universally across all stages of the disease, and regardless of its exact etiology. NPS lead to disability and reduced quality of life of PwD and their caregivers. NPS include hyperactivity (agitation and irritability), affective problems (anxiety and depression), psychosis (delusions and hallucinations), apathy, and sleep disturbances. Preclinical studies have shown that the orexin neuropeptide system modulates arousal and a wide range of behaviors *via* a network of axons projecting from the hypothalamus throughout almost the entire brain to multiple, even distant, regions. Orexin neurons integrate different types of incoming information (e.g., metabolic, circadian, sensory, emotional) and convert them into the required behavioral output coupled to the necessary arousal status. Here we present an overview of the behavioral domains influenced by the orexin system that may be relevant for the expression of some critical NPS in PwD. We also hypothesize on the potential effects of pharmacological interference with the orexin system in the context of NPS in PwD.

## Introduction

Dementia is characterized by a progressive decline in cognitive function and is a major cause of disability in the elderly. There are multiple underlying pathologic processes in dementia, including neurodegenerative and vascular diseases. Dementia affects different cognitive domains including memory, language, attention, and executive functions (1). Although cognitive impairment is the hallmark of dementia, behavioral problems, or neuropsychiatric symptoms (NPS), are commonly found across the entire severity spectrum of dementia. They include hyperactivity (agitation, irritability, disinhibition, aberrant motor behavior), psychosis (delusions and hallucinations), affective symptoms (depression and anxiety), apathy, euphoria, sleep disturbances and eating abnormalities. NPS are associated with accelerated disease progression, early institutionalization, and increased mortality. They reduce the quality of life of both PwD and their caregivers (2).

Treatment options for NPS consist of pharmacological and non-pharmacological interventions, with the latter being regarded as first-line option. There is a general shortage of approved drugs for the treatment of NPS in dementia (3), with so far only pimavanserin being approved for the treatment of psychosis in Parkinson Disease (4), and risperidone for the short-term treatment of aggression in Alzheimer Disease (in Canada and Europe) (2). The unmet need is huge, as shown by the number of drugs in clinical development for NPS in dementia (3). In clinical practice, the use of non-pharmacological interventions is restricted by the limited knowledge about their effectiveness and by the lack of specialized training for caregivers (5, 6). Due to these limitations and because severe NPS require quick resolution, NPS are often managed with off-label prescription of drugs (mostly antipsychotics, antidepressants, or anxiolytics) (6). This occurs despite the worrying adverse effects associated with most of these drugs (5).

The orexin system modulates a wide range of behaviors by integrating different types of information (e.g., metabolic, circadian, sensory, emotional) received from afferent neurons and by transforming these inputs into respective output signals distributed *via* a network of efferent axons projecting throughout the neuraxis. The orexin system comprises orexin (OX)-producing neurons that are located in a specific area of the dorso-lateral hypothalamus. OX neurons synthetize the peptide prepro-orexin, which is then cleaved to produce the two orexin peptides OX-A and OX-B (also named hypocretin-1 and hypocretin-2) (7). Axons of OX neurons project to a wide range of brain regions where OX-A and OX-B are then released (8) and bind to OX1 and OX2 receptors (OX1R, OX2R) to affect neuronal signaling (7). As such, the OX system modulates several homeostatic functions and behaviors.

Orexins were initially described to regulate feeding (9), but genetic and pharmacological studies later revealed that their primary role rather resided in the regulation of arousal and the sleep-wake cycle (10, 11). Ever since, understanding of the OX system's contribution to behavior, including many emotional and cognitive domains, has been expanding (8). The relative contribution of either OX1R or OX2R to the different functions of orexins is not fully elucidated, but pharmacological studies showed a more important role of OX2R signaling in wake promotion relative to OX1R (12). Drugs antagonizing orexin receptors have recently been developed and tested in clinical trials for various neuropsychiatric conditions. Dual orexin receptor antagonists (DORAs) are already approved for the treatment of insomnia (13).

We present here our perspective on which behavioral domains that are influenced by the OX system may be relevant for the expression of NPS in PwD. In this context we hypothesize that pharmacological modulation of orexin receptor signaling may ameliorate or aggravate selected NPS in PwD.

## Behaviors that are modulated by the orexin system

### Arousal and wakefulness

The most well-characterized function of the OX system is the regulation of sleep-wake transitions (14). Preclinical studies showed that experimental activation of OX neurons promotes and stabilizes wakefulness while their inhibition promotes non-rapid eye movement (non-REM) sleep (15–18) and that progressive loss of OX neurons in mouse models leads to fragmented wake (19). OX peptides exert their wake-stabilizing effects by interacting with the brain arousal network, which includes histaminergic, noradrenergic, and cholinergic neurons (14). Additionally, OX neurons support the wake state through promotion of muscle and motor tone (14, 20). Interestingly, the SAMP8 mouse model of accelerated aging show less non-REM and REM sleep and higher locomotor activity during the inactive phase of the light-dark cycle, which co-occur with higher cerebrospinal fluid (CSF) OX levels (21).

### Motor behavior

The orexin system contributes to the hypothalamic control of movement: brain injection of OX-A induces hyperlocomotion, stereotypies and grooming behavior in rats (22, 23); conversely, relative to wild-type mice, orexin-ablated mice show reduced exploratory activity under normal feeding conditions (24, 25). In addition, recent studies have shown that orexin cells are active during motion initiation (26). Their activation stimulates GAD65-expressing neurons in the lateral hypothalamus, which are then responsible for promoting normal locomotion and whose hyperactivity leads to hyperlocomotion (27).

### Aggressive behavior

**Figure 1:**
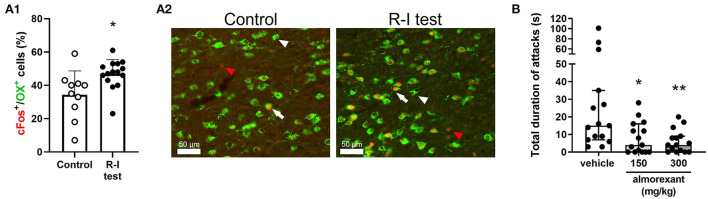
Implication of the OX system in reactive aggression in mice. **(A.1,A.2)** CD-1 mice were exposed to the resident-intruder (R-I) test or to control condition (i.e., staying in the home-cage, without any manipulation). The effect of aggressive behavior on activation of OX neurons was assessed using immunostaining. **(A.1)** Percentage of OX neurons expressing the immediate early gene and cellular activation marker c-Fos over the total number of OX neurons. Data are presented individually (scatter) and as mean (bars) + standard deviation (SD); *p < 0.05 vs. control, Student's *t*-test. **(A.2)** Representative image of OX^+^ cells (green) and c-Fos^+^ cells (red) in the lateral hypothalamus. Scale bar = 50 μm; white arrowhead: OX^+^ cell; red arrowhead: c-Fos^+^ cell; white arrow: OX^+^/c-Fos^+^ cell. **(B)** CD-1 mice were exposed to the R-I test after having been treated with vehicle or the dual orexin receptor antagonist almorexant, using a cross-over design. Data are presented individually (scatter) and as median with interquartile range. *p < 0.05, **p < 0.01 vs. vehicle, Dunn's multiple comparisons test (following repeated measure Friedman test). The methods used in these experiments are described in the supplementary information.

Reactive (defensive) aggression in rodents is largely controlled by hypothalamic circuitry (28). Activity of the ventrolateral portion of the ventromedial hypothalamus (VMHvl) is particularly associated with an aggression-related arousal state and execution of aggressive actions (29). OX neurons receive projections from the VMH *via* the dorsomedial hypothalamus (DMH) (30) and recruit GAD2-expressing neurons in the lateral habenula, whose activation promotes reactive aggressive behavior; this suggests that OX cells integrate information regarding aggression-related arousal (OX neurons become activated following the expression of aggressive behavior, see Figure 1A.1, A.2) and influence the activity of areas involved in the expression of reactive aggression. Drugs that antagonize both OX1/2Rs, or selectively OX2Rs, reduce reactive aggression in rodents (31–33) (Figure 1B). As mentioned above, SAMP8 mice have higher inactive phase CSF OX levels (21), and this strain shows higher reactive aggression relative to control animals (34, 35).

### Stress-reactivity and anxiety

The physiological and behavioral response to stressful environmental events allows animals to cope with situations relevant for survival. The hypothalamus is an integral part of the neurocircuitry coordinating the body's response to stress, and the contribution of the OX system has been largely investigated. Brain injection of OX peptides increases behavioral (e.g., grooming) and physiological (e.g., plasma levels of corticosterone) correlates of stress exposure (36, 37) and make mice agitated and hyper-responsive to sensory stimulation (38). Acute stressors recruit OX neurons (39, 40), and DORAs reduce stress-induced behavioral changes (41). In addition, brain infusion of OX peptides produces anxiogenic-like effects (42, 43), while OXR antagonists attenuate the expression of anxiety-like behaviors (44).

### Reward processing

Rodent studies indicate that the orexin system plays a role in the regulation of reward processing (7, 45, 46). OX neurons project to and modulate the activity of brain areas involved in reward-related behaviors including those of the mesocorticolimbic pathway and the brain's “hedonic hotspots” (8, 47). For example, optogenetic stimulation of OX-neurons-to-VTA projections increase the preference for and seeking of food rewards (48), and OXR antagonists reduce seeking and motivation for drugs of abuse (49, 50). In addition, OX signaling has been proposed to promote the motivational activation necessary for interaction with the environment and adaptive behaviors (51).

## Predicted effects of pharmacological orexin receptor modulation on NPS in PwD

### Background

The first drugs targeting orexin receptors demonstrating pharmacodynamic effects were two DORAs [namely, almorexant (ACT-078573) (11) and SB-649868 (52)]. Three DORAs are currently approved for the treatment of insomnia (i.e., suvorexant, lemborexant, daridorexant); furthermore, other psychiatric indications are also being investigated using DORAs or selective OX1R or OX2R antagonists (i.e., SO1RA or SO2RA) (13). OX2R agonists (SO2RAgs) with wake-promoting effects are in development for the treatment of narcolepsy (53, 54).

Different treatment regimens were tested in trials with OXR antagonists, depending on the targeted symptoms and the drug used. For example, in trials with DORAs/SO2RAs for insomnia and depression (55, 56) the drugs were administered at bedtime, and in a trial with SO1RA for binge eating disorder the drug was given during morning and evening meals (NCT04753164) (57). In the context of the possible use of OXR antagonists for the treatment of NPS in PwD, bedtime administration has the advantages of maximizing the drug's efficacy on nighttime problems and reducing the risk of daytime somnolence; however, little effect may be expected on disturbances that mostly manifest during the day [except those instances where amelioration of daytime symptoms is achieved through improved nighttime problems, as in the case of depression (56)]. Conversely, daytime dosing may have a greater impact on symptoms, which are expressed during the day, but (depending on the dose) it may be associated with a higher risk of somnolence. SO2RAgs, given in the morning to stabilize daytime wakefulness, may potentially also improve sleep at night by secondary means and thus normalize the disturbed wake-sleep rhythms observed in PwD.

### Sleep problems (insomnia, excessive daytime sleepiness)

PwD frequently present with difficulties in falling asleep (58), fragmented sleep (59), and excessive sleepiness during daytime (60). The sleep-wake disturbance in PwD leads to a progressive deterioration of circadian rhythm (61) and, in advanced stages of dementia, even to a reversal of the day-night sleep pattern (58). Sleep disturbance in PwD has been associated with the presence of other NPS such as anxiety and hyperactivity (62).

OXR antagonists administered at bedtime might have beneficial effects on sleep-related NPS in PwD. Indeed, two recent studies showed that sleep problems in AD can be ameliorated by DORAs (i.e., suvorexant:(63); lemborexant:(64)). Both trials showed improved sleep during the night. Actigraphy measurements in the lemborexant study demonstrated reduced sleep bouts during daytime, indicating a less disturbed sleep-wake rhythm as consequence of the nighttime treatment. Possibly due to the relatively long half-life of the drug, daytime somnolence was reported in the suvorexant trial, although this was not considered severe (65). Despite these beneficial effects on sleep parameters, effects of DORA on other nighttime NPS, including nighttime agitation, have not yet been assessed.

Given the wake-promoting effects of SO2RAgs, morning dosing may reduce the excessive daytime sleepiness observed in PwD and in addition help restore a normal sleep-wake rhythm.

### Hyperactivity (agitation/aggression, irritability, disinhibition, aberrant motor behavior) and anxiety

Agitation is one of the most frequent and pervasive NPS in PwD. Agitated behaviors are accompanied by signs of emotional distress and may include excessive motor activity (e.g., pacing, rocking, restlessness), verbal aggression (e.g., yelling, screaming), and/or physical aggression (e.g., grabbing, scratching, slamming doors) (66). Agitated behaviors can occur both during the day and the night (67), with nighttime agitation posing a huge burden for caregivers (68). Most aggressive behaviors shown by PwD can be interpreted as being reactive to environmental circumstances (e.g., lack of understanding of the caregivers' intentions, leading to rejection of care) (69). Other hyperactive behaviors include irritability and aberrant motor behavior, which may present as wandering, purposeless activity (e.g., insistently repeating demands or questions) and inappropriate activities (e.g., hiding objects in inappropriate places) (70). Anxiety is also highly prevalent in PwD (71), and it is expressed with both psychological and somatic manifestations (e.g., worry, palpitations, shortness of breath) (72).

From a neuropsychological perspective, hyperactive NPS and agitation, in particular, have been proposed to stem from an aberrant regulation of emotional salience, which may lead to overestimation of threat, hypervigilance and lowered stress thresholds (2).

As described above, the OX system regulates several behaviors of relevance for hyperactive NPS and anxiety. Specifically, OX system activation increases the motor output and stress reactivity, and OXR antagonists reduce reactive aggression, stress-induced behavioral changes, and anxiety in animals. Initial evidence in acute challenge tests in humans also indicate anxiolytic or stress-reducing capacity of SO1RAs or suvorexant (73–75). These laboratory findings suggest that an OXR antagonist might reduce hyperactive NPS and anxiety. One behavior that could benefit the most from bedtime administration would be nighttime agitation, because of the direct impact of the drug on both sleep and emotional reactivity.

Interestingly, the first beneficial effects of DORAs on hyperactive behaviors have been recently demonstrated in the case of nocturnal delirium. Delirium is a common condition in elderly and critically ill patients (76), characterized by a disturbance of consciousness which can be accompanied by hyperactive behaviors (77). Administration of suvorexant has been shown to improve nocturnal delirium in PwD and critically ill patients (76) (studies conducted only in Japan for the moment). These findings suggest that hyperactive behaviors may benefit from administration of DORAs; however, properly designed, controlled studies are needed to assess whether DORAs/SORAs improve hyperactive NPS in PwD. A clinical trial (NCT05307692) currently investigates the effects of seltorexant (a SO2RA) specifically on agitation as a primary outcome in patients with AD (78). Nobody has yet explored the effect of DORAs/SORAs on hyperactive NPS in the context of neuropsychiatric conditions other than PwD.

To the best of our knowledge, no trial has yet studied the effect of OXR antagonists on hyperactivity, when given during the day. Such timing of administration might attenuate hyperactive daytime symptoms but, depending on the level on OX2R antagonism exerted by the drug, could simultaneously also increase the daytime sleepiness.

### Apathy

Apathy is another NPS prevalent in PwD, which often presents itself already in the pre-dementia phase. Apathy is characterized by a lack of motivation, reduced interest in rewarding situations, and akinesia and has major impact on patients' quality of life (2). As mentioned above, preclinical studies showed that activation of the OX system positively regulates reward processing and motivational activation, and OXR antagonism reduces seeking of and motivation for rewards.

Given the blunting of reward perception by antagonism of orexin signaling, one can hypothesize that OXR antagonists may worsen the apathetic phenotype shown by PwD, especially if the drugs are administered at daytime, when patients engage and interact with their environment. However, in several neurodegenerative disorders, apathy is positively correlated with sleep problems and with low levels of activity during the day (79–81), raising the question of whether restoring the sleep-wake rhythm [i.e., by reducing sleep fragmentation and increasing daytime activity as shown by the lemborexant trial (64)] may have beneficial effects on apathy. Given that orexins promote motivational activation, daytime treatment with SO2RAgs could also alleviate apathetic behaviors.

## Discussion

Based on the preclinical knowledge on the function of the OX system, we believe that selected NPS experienced by PwD, including agitation/aggression, irritability, aberrant motor behavior, anxiety and sleep problems may be ameliorated by the pharmacological modulation of OXRs. At the same time, some NPS could also be negatively impacted by treatment with OXR modulators (e.g., daytime sleepiness, disturbed sleep-wake rhythm and apathy). Therefore, it is critical to choose the treatment regimen (i.e., bedtime vs. daytime), duration (i.e., acute vs. chronic) and the dose of the OX modulator wisely. Administration of OXR antagonists at bedtime, for instance, would have the clear advantage of ameliorating both sleep-wake disturbances and nighttime agitation. Secondary beneficial effects may then manifest on other NPS during the day as a consequence of a restored sleep-wake cycle: apathy and lack of engagement in daily activities, for example, may improve. Conversely, OXR antagonist administration during the day may have a more direct effect on reducing hyperactive NPS and anxiety. Although there is a risk of worsening sleep-wake problems and apathy, such risk could be mitigated by adopting an “as needed” (i.e., pro re nata, PRN) administration: for example, patients may be given an OXR antagonist only when they present with a presumably OXR modulator-responsive NPS (e.g., agitation). Different types of NPS may also require different degrees of OXR blockade or activation to be modulated. While studies suggested that at least 65% blockade of OX2R is necessary to promote sleep (12, 82), the level of OX1R/OX2R blockade/activation needed to modulate NPS-relevant behaviors is not known. Gathering more knowledge on this aspect may allow further fine-tuning of the treatment for a better efficacy-safety balance.

Identifying specific groups of PwD whose NPS are likely to respond to OXR modulation could be an elegant approach for a targeted treatment of NPS. This is challenging as NPS are not stable over time (83, 84) and several types of NPS often co-occur in PwD (85–87) [e.g., patients might present both apathy and agitation (88)].

SO2RAgs, although still very early in clinical development, should also be kept in mind as potential treatment that might become available in the future. A combination of OXR agonists, for instance, during the day, with OXR antagonists given during the night, might allow the successful treatment of the NPS spectra experienced by certain patients.

In the above considerations, one must consider the expected changes in the OX system itself that may occur in the context of neurodegeneration. Several studies have investigated OX levels in the cerebrospinal fluid (CSF) of PwD (especially in AD patients). Despite some evidence indicating higher OX levels in the cerebrospinal fluid (CSF) (89) and a positive correlation with the presence of NPS found in one study (90), a recent meta-analysis did not support the notion that CSF OX levels in AD patients differ from those of healthy controls (91). In apparent discrepancy to the lack of change in CSF OX levels, AD brains present with a 40-70% reduction in the number of OX neurons (73, 92, 93). These findings might be explained by a hyperactive phenotype of the remaining OX cells, induced by either cell-autonomous or circuit-related mechanisms, which may lead to higher OX production and release. Of note, a recent study showed that aged mice with more fragmented sleep, have a reduced number of OX neurons that are hyperexcitable due to impaired potassium channel currents (94), thus providing a mechanistic basis for the observed sleep disturbances. However, human evidence for a direct contribution of OX system dysregulation to NPS in PwD, is yet to be demonstrated.

In summary, there is increasing preclinical and emerging clinical evidence suggesting that the OX system is likely involved in the expression of several NPS in PwD. Pharmacological modulation of OXR signaling has the potential to improve certain NPS. The type of intervention (dual or selective OXR antagonism or agonism) and treatment regimen (daytime vs. nighttime administration; chronic vs. pro re nata) must be carefully chosen and adapted to the individual patient and symptomatology so as not to cause unwanted worsening of other NPS. We hope that future clinical studies will explore the efficacy and safety of OXR modulators in PwD.

## Data availability statement

The original contributions presented in the study are included in the article/Supplementary material, further inquiries can be directed to the corresponding author.

## Ethics statement

Experimental procedures were approved by the Basel-Landschaft Veterinary Office and adhered to Swiss federal regulations on animal experimentation.

## Author contributions

Conceptualization: GB, PC, and MS. Writing—original draft preparation: GB. Writing—review and editing: GB, PC, HM, and MS. Investigation and formal analysis: GB and HM. Supervision: MS. All authors contributed to the article and approved the submitted version.

## Conflict of interest

Authors GB, PC, HM, and MS are full-time employees of Idorsia Pharmaceuticals Ltd. The authors declare that this study received funding from Idorsia Pharmaceuticals Ltd., Allschwil, Switzerland. The funder had the following involvement with the study: conceptualization and investigation.

## Publisher's note

All claims expressed in this article are solely those of the authors and do not necessarily represent those of their affiliated organizations, or those of the publisher, the editors and the reviewers. Any product that may be evaluated in this article, or claim that may be made by its manufacturer, is not guaranteed or endorsed by the publisher.
