# Supplementary material for: What evidence is there for implicating the brain orexin system in neuropsychiatric symptoms in dementia?
Source: Front Psychiatry. 2022 Nov 25;13:1052233. doi: 10.3389/fpsyt.2022.1052233 (PMC9732550; doi:10.3389/fpsyt.2022.1052233)
Supplement: Supplementary file 1 [file Data_Sheet_1.docx]

**Supplementary information**

**Methods**

*Animals*

CD-1 male mice, purchased Janvier (Le Genest-Saint-Isle, France), were used as residents in the R-I test; they were 38-41 weeks old at the start of experiments and their average body weight was 50 g. Young, adult male C57BL/6J mice, purchased from Janvier, were used as intruders in the R-I test; they were 13-16 weeks old at the start of experiments and their average body weight was 35 g. All mice were maintained at the animal facility of Idorsia under standard lab conditions (temperature 20 ± 2 °C, relative humidity 55–70%, food and water ad libitum) under an inverted 12 h light–dark cycle (lights off 08:00 AM to 08:00 PM). Throughout the study, all mice were provided with enriching material in the home-cage: red transparent, plastic houses (Techniplast; Buguggiate, Italy), nesting material and wood sticks; enriching material was only removed during the R-I test. CD-1 mice were single-housed, while C57BL/6J mice were housed in pairs (with perforated transparent plastic divers to avoid fighting) in individually ventilated cages (IVCs) (GM500, Techniplast). Behavioral tests were conducted during the dark (active) phase under red light conditions to avoid disturbing the mice’ circadian cycle. Experimental procedures were approved by the Basel-Landschaft Veterinary Office and adhered to Swiss federal regulations on animal experimentation.

*Resident-intruder (R-I) test:*

Reactive aggression was evaluated using the R-I test, during which a single-housed male resident CD-1 mouse is confronted within its home-cage with a single, previously paired-housed, male intruder C57Bl/6J mouse for 10 min. Each R-I test session is video recorded, and aggressive behavior (i.e., physical attacks by the resident towards the intruder mouse involving direct physical contact) is scored off-line. The R-I test was conducted during the active phase of the mice (i.e., during the first half of the dark phase of the light-dark cycle), using red lights. Before conducting the R-I test session, CD-1 mice were exposed to 3 habituation R-I sessions. The interaction partners of the C57Bl/6J intruder mice were changed after each R-I session to avoid habituation/sensitization. The most aggressive CD-1 mice during the 3 habituation sessions were selected for the R-I test sessions.

- For the experiment assessing the impact of aggressive behavior on OX cell activity (Fig. 1A, panels 1, 2), on the day of R-I testing, 15 CD-1 mice were exposed to the R-I test and 10 CD-1 mice were used as controls (i.e., stayed in their home-cage, without any manipulation). 90 minutes after the end of the 10-min R-I test, resident mice received a lethal intraperitoneal injection of pentobarbital (150 mg/kg), their bodies were transcardially perfused with 10 mL ice-cold phosphate buffer saline (PBS; 0.1 M) containing heparin (1000 U/L) followed by 50 mL of 10% Neutral Buffered Formalin (Surgipath 3800598, Leica Biosystems). Brains were sampled and post-fixed for 24 h in 10 % formalin under mild agitation at 4°C, then transferred into PBS containing 0.01 % sodium azide and stored at 4°C until shipping to NeuroScience Associates Inc (NSA - Knoxville, TN, USA) for slicing and staining.
- For the experiment assessing the effect of DORA on reactive aggression (Fig. 1 B), 15 CD-1 mice were used and received treatments using a cross-over design (i.e., all mice receive all drugs in a counterbalanced manner). Three R-I test sessions were conducted until all CD-1 mice had received all three treatments: vehicle (methyl cellulose 0.25%/water) and the DORA almorexant at 150 and 300 mg/5ml/kg (administered per os, 1 h before testing). The time interval between each of the three R-I test sessions was 1 week. The total duration of attacks was manually and blindly scored off-line from videos recorded during the R-I test sessions.

*Staining and imaging:*

- Brains were coronally cut in cryo-sections at a thickness of 35 μm using the MultiBrain®/MultiCord® Technology (NSA), which allowed the simultaneous processing of all 25 brains, that were all embedded within the same matrix, at the same time. Brain sections including the lateral hypothalamus (LH) (-1.22 mm to -1.82 mm from bregma; at 210 μm intervals) were processed for c-Fos and orexin immunoreactivity. Free-floating sections were first stained with a primary rabbit polyclonal antibody for c-Fos (EnCor: RPCA-c-fos; 1:500) followed by a monovalent anti-rabbit F(ab) Cy3 fluorescent labelled secondary antibody (Jackson Labs: 711-167-003; 1:500); then, primary rabbit polyclonal antibody for orexin (Abcam: ab6214; 1:5000) followed by the corresponding fluorescent dye-labelled secondary antibody anti-rabbit Alexa Fluor 488 (Jackson Labs: 711-545-152; 1:500) were used. Fluorescence images were generated using an Olympus VS200 slide scanning system, equipped with a 20x objective lens. Sectioning, staining and imaging was conducted at NSA, and then the images were imported by Idorsia Pharmaceuticals and processed further.
- Images were analysed using Orbit Image Analysis, an open-source whole slide image analysis tool (1). The LH was identified on each stained brain section as defined by Franklin and Paxinos mouse brain atlas (2), and for each LH we measured the number of cells expressing OX (OX+ cells), c-Fos (c-Fos+ cells) and of those co-expressing OX and c-Fos (c-Fos+/OX+ cells). For each brain, 3-4 images were analysed as follows: cell counts for the right and left LH were summed and normalized to the total area (left + right LH) for each slide, and then the average values across all slides, were calculated to provide a single value for each animal for each of the parameters (i.e., OX^+^ cells, c-Fos^+^ cells, c-Fos^+^/OX^+^ cells). The % of c-Fos^+^/OX^+^ cells over the total number of OX^+^ cells was used for statistical analysis.

*Statistical analysis*

Analysis was performed with GraphPad Prism 8 software (GraphPad Software, Inc.). See the Figure legends for details.

**References**

1. Stritt M, Stalder AK, Vezzali E. Orbit Image Analysis: An open-source whole slide image analysis tool. PLoS Comput Biol. 2020;16(2):e1007313.

2. Franklin K, Paxinos G. The Mouse Brain in Stereotaxic Coordinates2001 2001//.
